# Inter- and intra-rater agreement among novices and comparison to an experienced consensus using the Gächter scale for the evaluation of septic joints

**DOI:** 10.3389/fvets.2025.1577046

**Published:** 2025-07-15

**Authors:** Tiffany I. Stockman, Michael P. Kowaleski, Jacqueline M. Hicks, Chanel N. Berns, W Brian Saunders, Robert J. McCarthy

**Affiliations:** ^1^Department of Clinical Sciences, Foster Hospital for Small Animals at Tufts Cummings School of Veterinary Medicine, North Grafton, MA, United States; ^2^Department of Biostatistics, Boston University School of Public Health, Boston, MA, United States; ^3^Department of Small Animal Clinical Sciences, Texas A&M University, College Station, TX, United States

**Keywords:** Gächter, septic arthritis, septic joint, arthroscopy, inter-rater agreement, intrarater agreement

## Abstract

**Introduction:**

The purpose of this retrospective study was to determine the inter-and intra-rater agreement among novice raters, as well as agreement between novice raters and an experienced consensus using the Gächter grading scale for the evaluation of the severity of septic joints in dogs.

**Methods:**

Three surgical residents served as novice raters, and two American College of Veterinary Surgery (ACVS) diplomates, experienced with arthroscopic evaluation of canine joints, served as the experienced consensus. Arthroscopy images were first evaluated by the experienced consensus and scored using the Gächter scale. After two supervised training sessions, novices applied the scale twice to the same images, 2 weeks apart.

**Results:**

The application of the Gächter grading scale was unreliable in dogs when utilized by novice raters.

**Discussion:**

Both the intra-rater agreement measured among the three novice raters and inter-rater reliability comparing the three novice raters to an expert consensus showed a consistently low concurrence among the individuals when tested at two separate time intervals. Lack of skill with arthroscopy, awareness of the anatomy and potential anatomic variations, and inadequate training in the application of the Gächter grading scheme could play a large part in a novice’s ability to apply the grading scale to a septic joint. Inter-rater agreement, while initially moderate, had a decreasing concurrence between the two-time intervals.

## 1 Introduction

Septic joints are only rarely reported in dogs, but their actual frequency is unknown (1). In humans, the reported incidence of septic arthritis varies from 2 to 5 cases/100,000 individuals annually in the general population, to 28–38 cases/100,000 individuals among patients with rheumatoid arthritis, and 40–68 cases/100,000 individuals annually among patients with joint prostheses (2, 3). Depending on the severity of infection and specific pathogen, sepsis can cause rapid and potentially permanent damage to the joint surface, with poor functional ability long-term (3–6). The most common etiologies reported in human medicine are immunocompromised patients, pre-existing joint diseases, the presence of joint prostheses, and/or joint surgery (3). In the veterinary field, common etiologies reported are a penetrating wound, a surrounding infection, or hematogenous spread (including skin infection and dental disease as the more common sources) (2).

Septic joints are generally suspected based on clinical findings, including fever, local hyperemia, joint effusion, lameness, and pain. Confirmation requires ancillary diagnostic testing such as joint fluid analysis, joint fluid culture, and/or synovial biopsy with tissue culture (1, 4). In humans, the gold standard treatment of septic arthritis is arthroscopic debridement and lavage in combination with appropriate antimicrobial therapy (3, 7).

In 1985, Andre Gächter described a classification system for human septic joints using arthroscopic findings to grade severity between I and IV (8). The Gächter scale can be applied to any joint disease, but in most cases has been applied to septic knee joints (6, 9). The use of Gächter scale grading has been demonstrated to have both prognostic and therapeutic significance in humans (6, 9). Similar information could be gleaned using the Gächter scale in veterinary patients, and possibly help direct clinical decision-making.

Intra- and inter-rater agreement is important for any diagnostic test to have value in clinical decision-making. Intra-rater is the degree of agreement among repeated sessions by the same rater, allowing assessment of the reliability of a single rater’s judgment. While inter-rater agreement is the degree of agreement among multiple raters who individually evaluate the same subject, it can assist in determining the consistency of assessment by different raters. The purpose of this study was to evaluate the inter-and intra-rater agreement of the Gächter grading scale when applied by novice raters, as well as compare agreement between novice raters and an expert consensus. Our hypothesis was that when applying the Gächter grading scale, novices would have a moderate intra-rater and inter-rater variability between the time periods, and good agreement with an expert consensus.

## 2 Materials and methods

### 2.1 Study design

Medical records from 2010 to 2022 from two surgical referral practices (XXX and XXX) were retrospectively searched for dogs diagnosed with septic joints. Inclusion criteria were a confirmed positive joint culture result (synovial fluid and/or synovial tissue biopsy and culture), availability of videos or still frames from arthroscopic assessment of the joint, and procedures performed by a board-certified surgeon or a resident under direct supervision of a board-certified surgeon. Additional data included age, breed, weight, physical examination findings, and the affected joint.

### 2.2 Arthroscopy image evaluation

Arthroscopy still frames and videos were assigned a random number (Random.org, Random Sequence Generator, Dublin, Ireland). Two American College of Veterinary Surgery (ACVS) diplomates, blinded to case data and experienced with arthroscopic evaluation of canine joints, initially reviewed all arthroscopy still frames and videos. Cases were excluded if they felt there was inadequate imaging quality for definitive evaluation. The remaining images were then graded based on the Gächter scale (Table 1), forming an expert consensus. The ACVS diplomates evaluated the videos and still frames together to form a consensus. There were several disagreements initially, but a discussion about key features allowed them to settle on an agreed-upon grade.

**Table 1:** Gächter grading scale.

Gächter scale	Description
I	Opacity of fluid Redness of the synovial membrane Possible petechiae No fibrin deposits
II	Severe inflammation Fibrinous deposition Purulent material
III	Thickening of the synovial membrane Cartilage erosion Compartment formation secondary to fibrin or purulent material
IV	Aggressive pannus infiltration of the cartilage Possible undermining of the cartilage Possible osseous erosions and cysts

**Figure 1:**
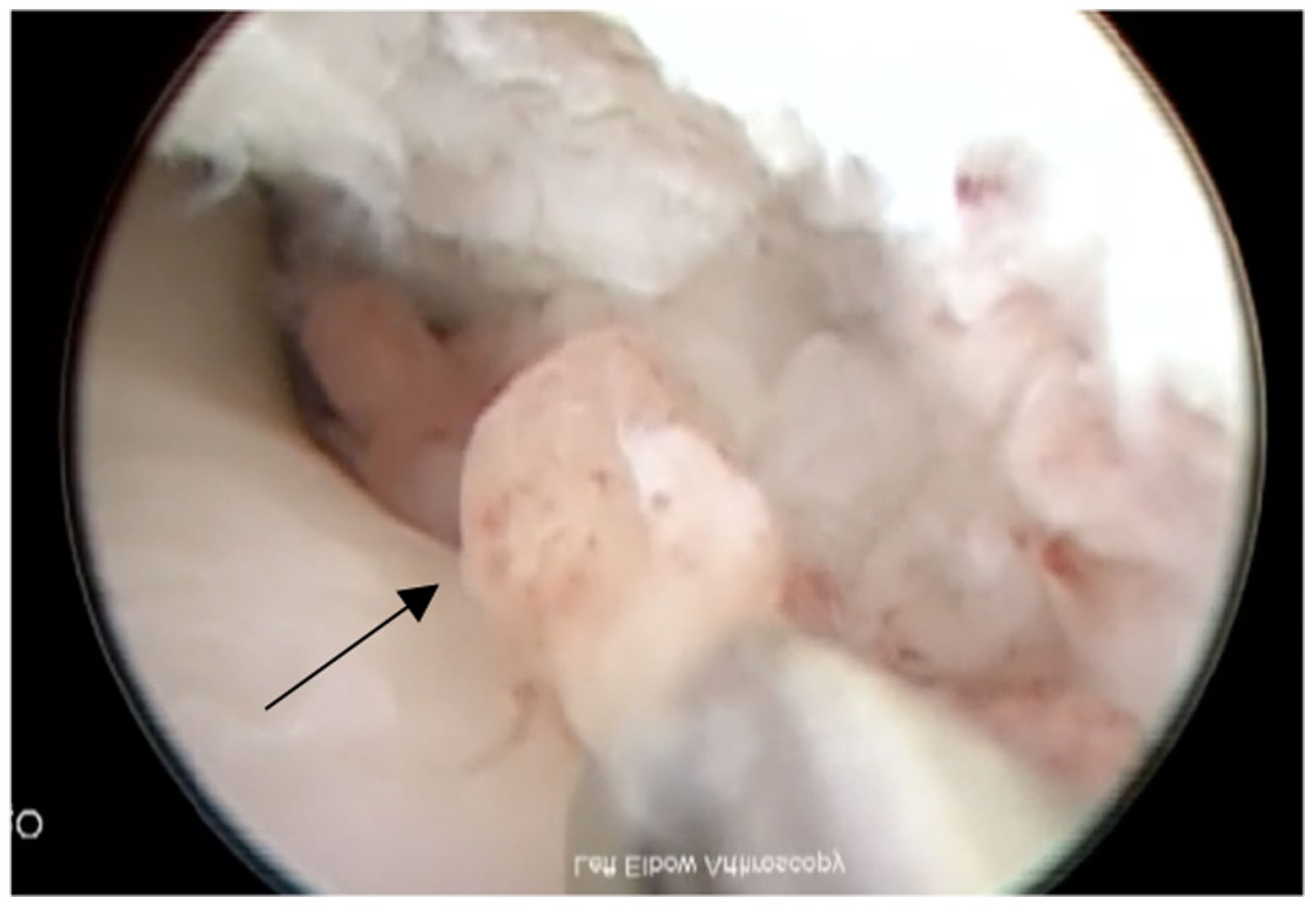
Example of grade I with petechiation of synovial membrane (shown with arrow).

**Figure 2:**
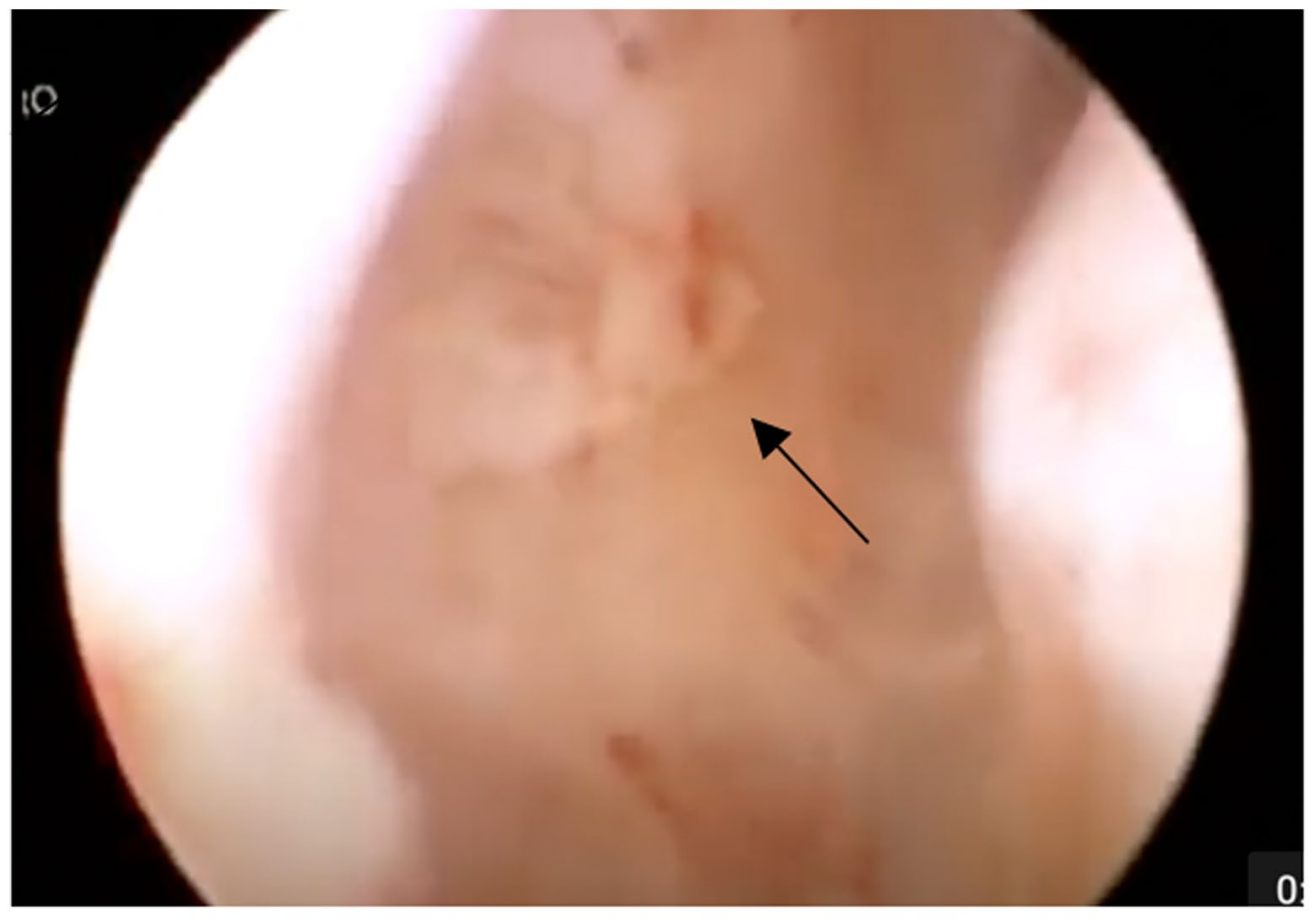
Example of grade II with fibrinous deposits (shown with arrow).

**Figure 3:**
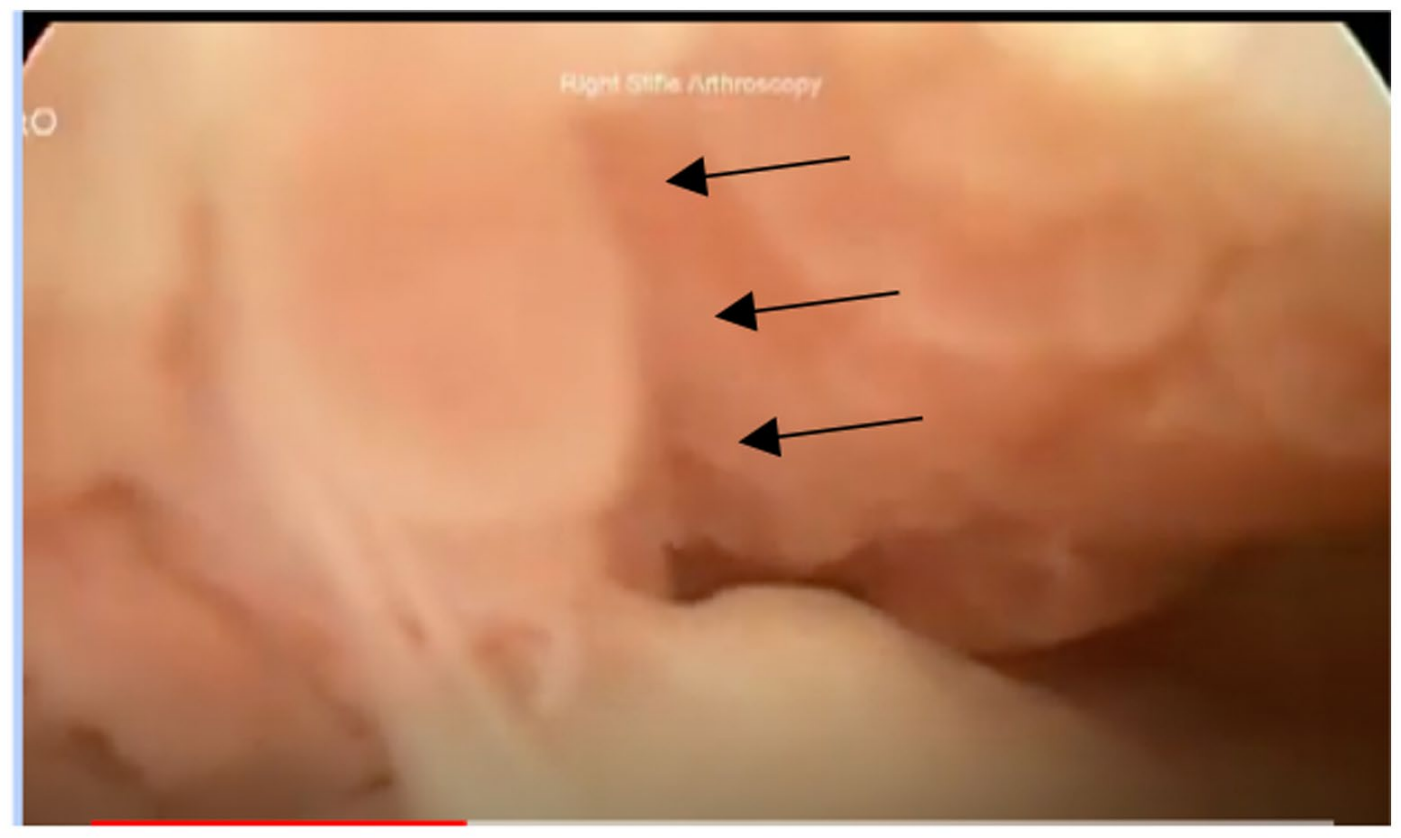
Example of grade III with compartment formation (outlined with arrows).

Three novice raters (ACVS residents) received two supervised training sessions in Gächter scale application in lecture format (approximately 60 min in length), followed by supervised evaluation of five arthroscopy cases to practice application of the Gächter scale. For actual study grading, novice raters were supplied with a description of the Gächter scale and example images (Figures 1–3). An image for grade IV was not included due to a lack of arthroscopic image availability in this case series. Novice raters used the Gächter scale to evaluate the same septic joint cases twice, 2 weeks apart. The images were presented in the same order for both grading sessions, with the cases including two videos, one video, and one still frame or two still frames. The primary author was not blinded and recorded all data.

### 2.3 Statistical analysis

The frequency and percentage describing the distribution of the rating scale in this sample were reported. To assess the inter-rater agreement and intra-rater agreement, we computed Light’s kappa coefficient and 95% confidence intervals based on an absolute agreement two-way mixed effects model where the subjects (dogs) and raters were modeled as random effects (10–12). All analyses were conducted using the “irr” and “DescTools” packages in RStudio (13–15). The statistical significance of the inter-and intra-rater agreement was examined; *p*-values less than 0.05 were considered statistically significant.

Kappa agreement was assessed, as listed in Table 2 (16). Any kappa below 0.60 indicates an inadequate agreement among the raters, and little confidence should be placed in the results. A negative kappa represents a disagreement, or an agreement worse than expected. A low negative value (0 to −0.10) indicates no agreement (16).

**Table 2:** Kappa agreement assessment.

Kappa range	Agreement assessment
< 0	No agreement
0–0.20	Minimal
0.21–0.39	Weak
0.40–0.59	Moderate
0.60–0.79	Strong
0.80–1.0	Almost perfect

From McHugh (16).

## 3 Results

### 3.1 Case data

Twenty-six cases were reviewed, with 18 meeting the final inclusion criteria; 8 cases were excluded by the expert consensus for inadequate imaging quality. Body weight of the final study population ranged from 30 to 78 kg (median: 43 kg), and age ranged from 2.5 years to 11 years (median: 5 years). Fourteen dogs were male (12 castrated, 2 intact) and 4 were female (3 spayed, 1 intact). Breeds included Labrador Retriever (*n* = 4, 22%), German Shepherd (*n* = 3, 17%), Mastiff (*n* = 3, 17%), Newfoundland (*n* = 2, 11%), Saint Bernard (*n* = 2, 11%), Mixed Breed (*n* = 2, 11%), Bernese Mountain Dog (*n* = 1, 5.5%), and Boxer (*n* = 1, 5.5%). The joint most affected was the stifle (*n* = 14, 78%), followed by the elbow (*n* = 3, 17%) and hip (*n* = 1, 5%). The cause for the majority of cases was not known or recorded and thus not evaluated for this study.

Arthroscopic images consisted of still frames (*n* = 6, 33%), videos (*n* = 11, 61%), or a combination of both (*n* = 1, 6%). Each case included either two still frames, one still frame and one video, or two videos. The videos ranged from 8 to 41 s in length. Organisms cultured from infected joints included *Staphylococcus pseudintermedius* (*n* = 8, 44%), *Beta-hemolytic streptococcus* (*n* = 7, 38%), *Pasteurella* (*n* = 1, 6%), *Staphylococcus schleiferi* (*n* = 1, 6%), and *Actinomyces* (*n* = 1, 6%). All joints were reported to be infected with a single organism.

### 3.2 Arthroscopic evaluation

The results of the expert consensus and novice evaluations at both intervals are shown in Table 3. The expert consensus classified 5.6% (*n* = 1) as grade 1, 88.9% (*n* = 16) as grade 2, 5.6% (*n* = 1) as grade 3, and 0% (*n* = 0) as grade 4.

**Table 3:** Grades assigned to 18 septic joints utilizing the Gächter grading scale for both novice raters and an expert consensus at two-time intervals.

	Grade 1	Grade 2	Grade 3	Grade 4
Expert consensus	1 (5.5%)	16 (89%)	1 (5.5%)	0 (0%)
T1				
Rater 1	5 (28%)	7 (39%)	4 (22%)	2 (11%)
Rater 2	9 (50%)	5 (28%)	2 (11%)	2 (11%)
Rater 3	4 (22%)	12 (67%)	1 (5.5%)	1 (5.5%)
Totals (54)	18 (33%)	24 (44%)	7 (13%)	5 (9%)
T2				
Rater 1	5 (28%)	8 (44%)	3 (17%)	2 (11%)
Rater 2	8 (44%)	8 (44%)	1 (6%)	1 (6%)
Rater 3	4 (22%)	10 (56%)	3 (16%)	1 (6%)
Totals (54)	17 (31%)	26 (48%)	7 (13%)	4 (7%)

T1, time interval 1; T2, time interval 2.

The results of the inter-rater and intra-rater agreement between novices and the expert consensus at both intervals are shown in Table 4.

**Table 4:** Inter- and intra-rater agreement values between 3 novice raters and an expert consensus for 18 dogs using the Gächter scale grading scale.

	Light’s kappa	Kappa agreement assessment	*P*-value
Inter-rater			
Between novices at T1	0.413 (0.348, 0.477) *	Moderate	<. 0001
Between novices at T2	0.294 (0.092, 0.495) *	Weak	0.004
Between novice and expert at T1	0.248 (−0.387, 0.882) *	Weak	0.444
Between Novice and expert at T2	0.168 (−0.587, 0.922) *	Weak	0.663
Intra-rater			
Novice 1	0.268 (0.88) **	Weak	0.517
Novice 2	0.309 (0.80) **	Weak	0.359
Novice 3	0.631 (0.41) **	Strong	0.001

*CI, confidence interval. **SEM, standard error of the mean. T1, time interval 1, T2, time interval 2; parenthesis, represents low, high, or mean values.

## 4 Discussion

The results of this study indicate that the application of the Gächter grading scale was unreliable in this study in dogs when applied by novice raters to pre-recorded videos and still-frame images. Both the intra-rater agreement measured among the three novice raters and comparison to an expert consensus showed a consistently low concurrence among the individuals when tested at two separate time intervals. Inter-rater agreement, while initially moderate, had a decreasing concurrence between the two time intervals. Our hypothesis that the Gächter grading scale would show sufficient agreement to be used by inexperienced surgeons was incorrect based on the results obtained during this study.

Intra-rater agreement among novices between time intervals was moderate (0.402), suggesting that frequent review of the grading scale and criteria for each stage should be performed until the rater is comfortable with applying the scale. Several studies have confirmed the novice learning curve among surgery residents, with improved performance after lecture, live demonstration by senior surgeons, and hands-on training (17, 18). Residents performing their first 100 surgeries were more than three times more likely to encounter complications compared with residents who had performed at least 600 surgeries. Intra-rater agreement could potentially improve with further training from experienced clinicians and increased frequency of application (19).

Inter-rater agreement was initially moderate (0.413), but nearly halved (0.294) between the time periods evaluated. Before application, the novices had two lecture-style training sessions (approximately 60 min in length) with case example discussion. These training sessions were 1 week apart, followed by the novice group being provided five cases for individual grading that were returned within 7 days (20, 21). The five cases provided were not utilized in the final grading. Inter-rater agreement is critical for a diagnostic test to be useful and is commonly assessed in other tests in veterinary medicine to ensure reliability (22). A lack of skill with arthroscopy and awareness of the anatomy and potential anatomic variations could play a large part in a novice’s ability to apply the grading scale to a septic joint. Since there is no comparable grading scale in the veterinary field, introducing this new concept may be difficult for novices inexperienced with arthroscopy.

Of the 18 cases evaluated, 16 were considered grade 2 by the expert consensus. A meta-analysis in humans of 318 total septic joints indicated that 75 cases were grade 1 (24%), 157 cases (49%) were grade 2, 69 cases were grade 3 (22%), and 17 cases were grade 4 (5%) (6). Cases that were grade 1 and 2 occurred when the interval time between the appearance of symptoms and the treatment was approximately 7–15 days (7). Regardless of the procedure performed, prompt intervention is the most critical factor for eradicating infection and achieving good clinical outcomes (23). Infection within the joint and synovial membrane results in an inflammatory response. This releases destructive enzymes, and since synovial fluid is rich in nutrients, the fluid therefore makes an excellent growth medium for infection (24). Studies performed on rabbit models established that irreversible damage could occur to cartilage, bone, capsule, and ligamentous structures without intervention within 5 days of infection (23). While the time to intervention after the onset of clinical disease was not assessed in the present study, rapid intervention may have contributed to the majority of cases being classified as relatively mild grade 2 on the Gächter scale.

Large-breed, middle-aged male dogs were most frequently represented here, consistent with previous studies (25). Like previous reports in both humans and dogs, the stifle was most affected (6, 24). While an explanation for stifle predilection has not been reported, previous surgery or pre-existing joint disease, such as osteoarthritis, which frequently occurs in large or giant breed dogs, increases the risk of developing septic arthritis (24, 26–28). Future studies should document any pre-existing conditions that affect the joint. In the current study, all joints were infected with a single bacterium; however, a variety of organisms were cultured across cases. This is consistent with previously reported studies, but it does not eliminate the risk of multiple organisms affecting a joint (4, 26).

Several limitations exist in the current study. A larger sample size would have been preferred, but septic joints are infrequent, and several cases were eliminated because of a lack of positive culture. A joint can be septic without exhibiting a positive culture, which can further limit the number of available cases for study (4, 29). A larger sample size would also allow for more variety in the grades, as the limitation within the current study is the lack of variability, with the majority being grade 2. Arthroscopy imaging for this study was not obtained for the application of the Gächter grading scale originally; therefore, specifically recorded imaging was not available for the presence or absence of specific lesions to which the grading scale could be applied. If arthroscopy imaging was obtained with the application of the Gächter grading scale in mind, the outcome might have been different. Cases were also eliminated due to poor-quality images obtained with arthroscopy. Obtaining quality images with arthroscopy is necessary for the rater to identify redness of the synovial membrane, fibrinous deposits, purulent material, cartilage erosion, and/or pannus, required to accurately apply the Gächter grading scale. There was no subjective difference in the ability to assign a grade based on the availability of video versus still-frame. For consistency, future studies may consider a preference for using one modality for the entire study. A final limitation is the number of evaluations performed by novice raters. A more definitive pattern could be determined if additional evaluations were performed, showing that raters progressively worsened (or improved) in application in this study.

Future studies should consider inter-rater and intra-rater agreement among experienced raters to determine if the low agreement of the Gächter grading scale demonstrated in this study is due to experience level or if the Gächter grading scale is difficult to apply to the veterinary field, even for experienced clinicians. During the development of the experienced consensus, an agreed-upon consensus grade was subjectively reached very quickly and easily between the two experienced surgeons. It is the authors’ opinion that the application of the Gächter scale is possible, but likely requires experienced observers and appropriate training, and could be assessed in a future study. The observation that inter-rater agreement was initially moderate and nearly halved between the time periods evaluated may indicate that more rigorous training and experience with the grading system prior to applying it may improve the outcome. Further considerations could include a larger case number to assess the trends of grades, additional time periods, different training methods, and assessment of the value of the Gächter classification in predicting the clinical outcome of veterinary patients.

In conclusion, while the Gächter classification has shown to be a valuable prognostic tool in predicting the outcome of surgical treatment of septic joints in humans, a significant variability between the novice raters and the lack of agreement with the experienced consensus in the current study suggests that additional training time or alternate methods of training are warranted prior to the routine application in veterinary patients.

## Data Availability

The original contributions presented in the study are included in the article/supplementary material, further inquiries can be directed to the corresponding author.
